# Telomere shortening leads to earlier age of onset in ALS mice

**DOI:** 10.18632/aging.100904

**Published:** 2016-02-24

**Authors:** Birgit Linkus, Diana Wiesner, Martina MeΔner, Alexander Karabatsiakis, Annika Scheffold, K. Lenhard Rudolph, Dietmar R. Thal, Jochen H. Weishaupt, Albert C. Ludolph, Karin M. Danzer

**Affiliations:** ^1^ Department of Neurology, Ulm University, 89081 Ulm, Germany; ^2^ Department of Clinical & Biological Psychology, Ulm University, 89081 Ulm, Germany; ^3^ Department of Internal Medicine III, Ulm University, 89081 Ulm, Germany; ^4^ Leibniz Institute for Age Research, 07745 Jena, Germany; ^5^ Department of Pathology, Ulm University, 89081 Ulm, Germany

**Keywords:** telomere length, amyotrophic lateral sclerosis, telomerase dysfunction, astroglia, microglia

## Abstract

Telomere shortening has been linked to a variety of neurodegenerative diseases. Recent evidence suggests that reduced telomerase expression results in shorter telomeres in leukocytes from sporadic patients with amyotrophic lateral sclerosis (ALS) compared with healthy controls. Here, we have characterized telomere length in microglia, astroglia and neurons in human post mortem brain tissue from ALS patients and healthy controls. Moreover, we studied the consequences of telomerase deletion in a genetic mouse model for ALS. We found a trend towards longer telomeres in microglia in the brains of ALS patients compared to non-neurologic controls. Knockout of telomerase leading to telomere shortening accelerated the ALS phenotype in *SOD1^G93A^*–transgenic mice. Our results suggest that telomerase dysfunction might contribute to the age-related risk for ALS.

## INTRODUCTION

Telomeres are the distal ends of chromosomal DNA consisting of repetitive DNA sequences that do not encode any gene product [1]. Their main function is to cap chromosomal ends to protect them from being recognized as broken DNA, thus preventing their degradation and participation in fusion events [2]. Therefore telomeres are essential for maintenance of genomic stability [1, 3, 4]. Because of the inability of complete DNA duplication at the chromosome ends telomere length becomes progressively shorter after repeated cell divisions [5]. Thus, telomere shortening is regarded to represent cellular aging. Indeed in all human tissues telomere shortening has been shown with age, except for the brain and myocardium [6]. The adult brain is considered to be in a stable condition since glial cells are replaced very slowly under normal conditions and neurons in adults are postmitotic maintaining a constant telomere length [7-9].

Telomere shortening has been shown to be associated with various age-related diseases including hyper-tension, arterial stiffening and atherosclerosis [10-14]. Also the risk for several neurodegenerative diseases including Alzheimer's disease, dementia with Lewy bodies and Parkinson's disease has been associated with telomere shortening in leukocytes [13, 15, 16, 17]. In addition, a link between telomere shortening and physical or chronic mental stress [18, 19], smoking, obesity and diabetes mellitus has been reported [13, 20-22]. Besides age one common denominator of many of the above mentioned diseases is increased oxidative stress. In fact, several studies indicate that increased oxidative stress accelerates the attrition of telomeres [23-25].

Amyotrophic lateral sclerosis (ALS) is an age-related neurodegenerative terminal disease involving the progressive degeneration of neurons within the motor cortex, brainstem and spinal cord [26, 27]. Although its pathogenic mechanism is uncertain, oxidative stress is believed to be implicated in its pathology [28-36]. Due to the severity of the disease and missing therapeutic options, pathological psychological stress often accompanies ALS [37-39]. Interestingly, a recent study identified shorter telomeres in leukocytes from sporadic ALS patients compared with healthy controls [40]. However, telomere length especially in human ALS brains has not been characterized yet.

Telomere shortening can be compensated by the enzyme telomerase, which is capable to synthesize telomeres *de novo* [41]. Therefore telomerase knockout mice (mTerc^−/−^) provide an elegant experimental system to study aging induced by telomere dysfunction and DNA damage [42-44]. Interestingly, a strong reduction in telomerase expression in spinal cord of sporadic ALS patients could be observed compared to healthy controls [40].

Together, existing data from the literature point to a role for telomere dysfunction in ALS. The aim of this study was to characterize telomere length in human brains from ALS patients and and healthy controls and to determine the consequences of telomere dysfunction in ALS SOD1^G93A^ mice.

## RESULTS

### Comparison of telomere length in different cell types in human ALS and control brains

**Figure 1:**
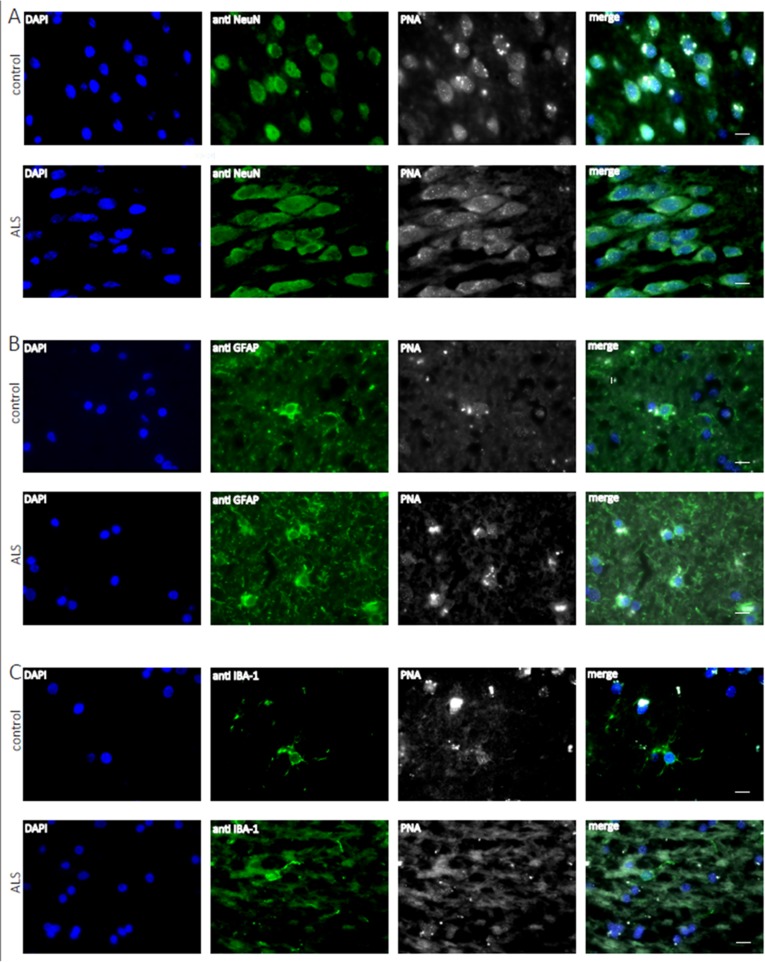
Telomere length analysis of human hippocampal ALS and control tissue at the cellular level Representative fluorescence images from the hippocampus from ALS patients and non-neurologic controls showing telomere specific PNA-probe Cy3-OO (CCCTAAA)_3_ (white), DAPI staining (blue) and in green NeuN positive neurons (**A**), astroglia positive GFAP cells (**B**) and microglia positive Iba I cells (**C**).

**Figure 2:**
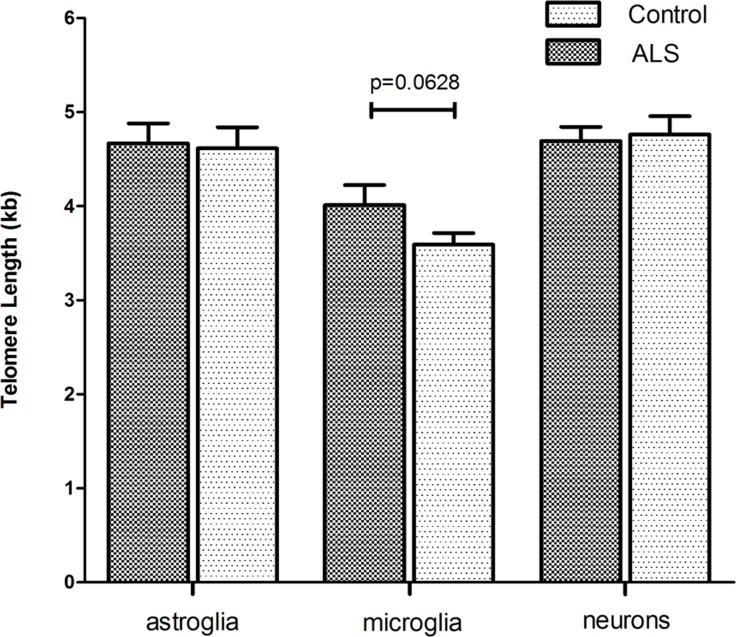
Cell type specific telomere length in hippocampal tissue of ALS- patients and non-neurological control-individuals Telomere fluorescence intensity (TFI) of astroglia, microglia and neurons was measured using a combined protocol for qFISH and immunhistochemistry. Subsequently TFI was converted into Telomere length (TL) using a linear equitation resulting from qFISH and linear regression analysis of five selected cell lines with defined and stable telomere length. Values represent the mean ± SEM. P-values are sex- and age- adjusted based on an appropriate multiple linear regression model.

Accumulating evidence has shown that inflammatory processes participate in the pathogenesis of ALS. In particular microglia and astroglia are believed to play a key role in ALS pathogenesis [45]. Since ALS is an age related disease, we asked whether telomere shortening would affect microglial and astroglial cells in human ALS brains (a detailed description of the patient cohort can be found as Supplementary Table 1). Additionally, we also included neurons in the analysis to investigate whether telomere shortening would affect the maintenance of postmitotic neurons in ALS. To determine telomere length in those different brain cell types quantitative fluorescence in situ hybridization (qFISH) was performed. For cell type specific telomere fluorescence intensities (TFIs) of neurons, astroglia and microglia a combined staining protocol of qFISH and antibody staining with a fluorescent secondary antibody was used. We also included an external calibration method using five different tumor cell lines which maintain a defined and known telomere length distribution [46] determined by Southern Blot analysis (Supplemental Table 2). To avoid day to day variations and to be able to convert TFIs into kilobases a standard curve was generated by simultaneous qFISH analysis of tumor cell lines and human tissue. Representative fluorescence images from neurons, astroglia and microglia in human ALS brains and non-neurologic controls using qFISH are demonstrated in Fig. 1 A-C. After conversion of TFIs into telomere length in kilobases we found no difference in telomere length in neurons and astrocytes between ALS patients and non-neurological controls. Surprisingly, in microglia we found a trend towards longer telomeres in ALS patients compared to non-neurologic controls, although statistical significance was not reached (Fig. 2).

### Telomere shortening accelerated age of onset and reduces survival in SOD1(G93A) mice

To further characterize the role of telomere length in ALS we turned to a well-characterized transgenic model of mice over-expressing the human ALS-associated SOD1 G93A mutation (SOD1^G93A^-mice) [47] and crossed through three generations with telomerase knockout mice (mTerc^−/−^) (Supplement Figure 1). The following cohorts were used: *G4 mTerc^−/−^;SOD1^G93A^*; *mTerc^+/+^;SOD1^G93A^* (hereafter referred as *SOD1^G93A^*). Telomere shortening in mTerc^−/−^ in the fourth generation was described previously [43].

**Figure 3:**
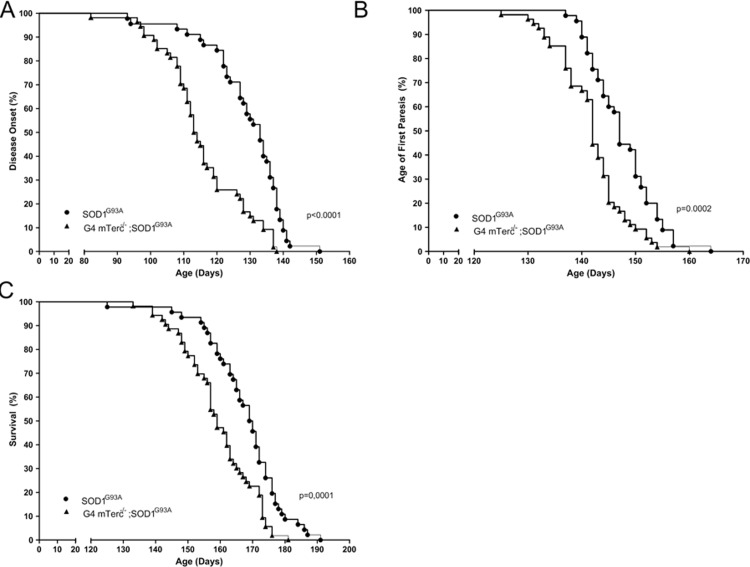
Telomerase deficiency affects disease course of SOD1^G93A^-transgenic mice (**A**) Kaplan-Meier plot of disease onset. *G4 mTerc^−/−^;SOD1^G93A^* mice show a 13 day earlier disease onset compared to SOD1^G93A^-mice. Disease onset: *G4 mTerc^−/−^;SOD1^G93A^-*mice 116 days ± 12.256, *SOD1^G93A^-*mice 129 ± 11.800. (**B**) Kaplan-Meier plot of age of fist paresis. *G4 mTerc^−/−^;SOD1^G93A^* mice with telomerase deficiency show hindlimb-paresis 6 day earlier compared to SOD1^G93A^-mice. Age of fist paresis: *G4 mTerc^−/−^;SOD1^G93A^-*mice 142 days ± 6.482, *SOD1^G93A^-*mice 148 ± 5.958. (**C**) Kaplan-Meier plot of survival. Survival of *G4 mTerc^−/−^;SOD1^G93A^* mice with telomerase deficiency is reduced about 9 days compared to SOD1^G93A^-mice. Survival: *G4 mTerc^−/−^;SOD1^G93A^-*mice 160 days ± 10.995, *SOD1^G93A^-*mice 169 ± 10.060. *G4 mTerc ^−/−^;SOD1^G93A^* (n= 54), *SOD1^G93A^* (n=47). Mice of both genders were monitored daily.

To determine the impact of telomere shortening on ALS-associated motor symptoms in *SOD1^G93A^* we compared disease onset, first signs of paresis, and survival in *G4 mTerc^−/−^;SOD1^G93A^* and single transgenic *SOD1^G93A^*. We found in double transgenic *G4 mTerc^−/−^;SOD1^G93A^* an 13 day earlier disease onset than in single transgenic *SOD1^G93A^* mice (*mTerc^−/−^;SOD1^G93A^-*mice 116 days ± 12.256, *SOD1^G93A^-*mice 129 ± 11.80) (Fig. 3A). In line with this observation, we also found in *G4 mTerc^−/−^;SOD1^G93A^* mice signs of first paresis six days earlier compared to single transgenic *SOD1^G93A^* mice (*G4 mTerc^−/−^;SOD1^G93A^-*mice 142 days ± 6.48, *SOD1^G93A^-*mice 148 ± 5.96) (Fig. 3B). Most importantly, also lifespan was significantly reduced in *G4 mTerc^−/−^;SOD1^G93A^* mice compared to *SOD1^G93A^* mice (*G4 mTerc^−/−^;SOD1^G93A^*-mice 160 days ± 10.99, *SOD1^G93A^*-mice 169 ± 10.06) (Fig. 3C). The influence of telomere shortening on disease onset, age of first hind limb paresis and survival was also seen when we performed gender specific analysis (data not shown).

Together, these results suggest that telomere dysfunction deteriorates ALS phenotype leading to earlier age of disease onset and death in *SOD1^G93A^* mice.

## DISCUSSION

The present study provides evidence that telomere dysfunction contributes to ALS pathogenesis. First we found that microglial cells in the brains of ALS patients exhibit a trend toward longer telomeres compared to non-neurological controls. Vice versa, knockout of telomerase leading to telomere shortening was capable to deteriorate the ALS phenotype in *SOD1^G93A^* -mice. Recent studies have linked telomere dysfunction to a variety of neurological diseases. Significantly shorter leukocyte telomeres have been reported in Alzheimer's disease patients as compared with age matched healthy controls [48]. Telomere shortening could also be observed in leukocytes of patients with dementia with Lewy bodies when compared to non-neurological controls [15]. An association between telomere shortening and the risk for Parkinson's disease has also been reported in several studies [16, 17, 49]. However, there are also studies reporting no such association in PD [50, 51]. Moreover, a recent study demonstrates decreased expression of human telomerase reverse transcriptase (hTERT) in leukocytes of ALS patients and consequently shorter telomeres in ALS leukocytes [40]. In contrast, we found a trend for increased telomere length in microglia in the brains of ALS patients. This seems contradictory at first, however, increased telomere length can be observed as an enhanced telomere stabilization mechanism when the proliferative capacity is increased. However, the trend towards telomere elongation in microglial cells in the brains of ALS patients could represent a mechanism compensating the increased proliferation of microglial cells in ALS brains. Indeed increased telomerase activity and telomere elongation could be observed in microglia after facial nerve axotomy [52]. Importantly, microglia retain a robust proliferative potential, especially under conditions of CNS injury involving neuronal damage or cell death [53-56]. Flanary et al. could demonstrate that microglia utilize telomerase to regulate telomere length in vivo and that the increase in microglial telomere length in axotomized facial nuclei is likely to compensate for telomere shortening that would otherwise occur [52]. We thus hypothesize that the trend towards elongated microglial telomeres in the brains of ALS patients is linked to the higher proliferative activity of this cell type, and thus a pre-requisite for microglial functions in ALS. Microglial cells have been implicated in ALS disease progression [57], although their exact role, especially with regard to ALS onset, is still controversially discussed.

Thus, in order to further elucidate the functional relevance of our findings on telomere length in human ALS brain, we investigated whether telomerase knockout would affect ALS pathogenesis in the *SOD1^G93A^* ALS mouse model. We found that telomerase knockout resulting in telomere shortening is capable of reducing survival and acceleration of ALS disease onset in *SOD1^G93A^* mice. Interestingly, a decrease of hTERT expression leading to reduced telomerase activity was recently reported in post-mortem spinal cord of ALS patients compared to healthy controls [40]. Low levels of hTERT in mature neurons might contribute to their vulnerability of various oxidative insults [58, 59]. In contrast, it has been suggested that telomerase induction might represent an endogenous compensatory mecha-nism to protect against ischemic injury in the brain [60].

Moreover, our results complement the results of Eitan et al. who showed that a novel compound, AGS-499, that increases telomerase activity and expression in the mouse brain and spinal cord, delays ALS disease onset and progression in transgenic *SOD1^G93A^* mice [61].

In conclusion, we could not confirm a reduction in telomere length in the post-mortem brain tissue of ALS patients, in contrast to previous findings in ALS patient leukocytes [40]. The mild trend towards longer telomeres in microglial cells is most likely related to their known proliferation in affected ALS brain tissue, and might thus play a role in the microglial contribution to ALS disease progression. Considering the earlier and enhanced phenotype of mutant *SOD1^G93A^* transgenic mice lacking telomerase activity combined with the fact that telomeres shorten during aging further supports that shorter telomeres could contribute to the usually age-related disease ALS.

Restoration of telomere function or other components of the telomeric complex might thus be an approach to attenuate neurodegeneration in ALS or other age-related neurologic disorders.

## METHODS

### Animals

Male and Female double mutant G4 *mTerc^−/−^*;*SOD1^G93A^-* and hemizygous *SOD1^G93A^*-mice were used in this study. Double mutant *SOD1^G93A^; mTerc^−/−^* of the fourth generation were generated by multiple steps of intercrosses. In a first step *SOD1^G93A^* males (B6.Cg-Tg(SOD1-G93A)1Gur, stock #004435, the Jackson Laboratory) were mated with heterozygous *mTerc*^+/−^-females (B6.Cg-*Terc^tm1Rdp^*/J, stock #004132, the Jackson Laboratory). In a second step *mTerc^+/−^*-females were mated with *mTerc^+/−^;SOD1^G93A^-*males to generate *SOD1^G93A^-* mice lacking telomerase activity *(G1 mTerc^−/−^;SOD1^G93A^*- mice). Since early generation mice that are homozygous null for the Terc gene are phenotypically normal we crossed *G1 mTerc^−/−^*- females with *G1 mTerc^−/−^;SOD1^G93A^*- males in a third breeding step. Two additional intercrosses of (identical to the third step) were necessary to generate *G4 mTerc^−/−^;SOD1^G93A^*- mice. Hemizygous *SOD1^G93A^*- control mice (stock #004435, the Jackson Laboratory) came from a separate mice cohort. All animals were housed in humidity-, temperature- and light controlled animal facility with ad libitum access to standard chow and water. Animal procedures followed current EU regulations (Reg O117).

### Genotyping

*SOD1^G93A^*- mice were identified by DNA tail genotyping as described previously [62]. For genotyping of heterozygous and homozygous *mTerc^−/−^* mice a Multiplex PCR reaction was applied using the following primers. mTRR (TTCTGACCACCACCAA CTTCAAT), 5PPgK (GGGGCTGCTAAAGGGCAT) and mTRWtF (CTAAGCCGGCACTCCTTACAAG); the size of the amplified wildtype DNA fragment is 250 bp and 180 bp of the knockout DNA fragment.

### Disease onset, first paresis and survival

For evaluation of the disease course three different stages of the disease were assessed in G4 *mTerc^−/−^*;*SOD1^G93A^-* and hemizygous *SOD1^G93A^*- mice. Asymptomatic mice show normal gait and no hindlimb paresis. Disease onset was determined when gait impairment became visible, but no affection of hindlimbs was detectable. First paresis was defined when of at least one hindlimb was affected. For survival analysis mice were sacrificed when they reached the end stage of the disease which is defined as the inability to rise immediately after being placed on the side [63].

### Human samples

The brain samples were received from the brain bank of Ulm University. All human experiments were performed in accordance with the declaration of Helsinki and have been reviewed and approved by the Local Research Ethics Committee. For this study hippocampal sections from 24 autopsy cases (11 ALS and 13 non-neurologic control subjects) were studied (for study patient cohort see Supplemental Table 1). All patients with ALS fulfilled the El-Escorial criteria for definite ALS diagnosis and were confirmed by autopsy. All ALS cases used in this study showed TDP-43 inclusions in the spinal cord and in the central or frontal cortex. Pathological ALS stages were assessed according to Brettschneider et al. [26]. Tissues obtained at autopsy were fixed in a 4% aqueous formaldehyde solution and embedded in paraffin using standard protocols.

### Assessment of telomere length in different cell types

For cell type specific Telomere Fluorescence Intensity (TFI) measurement of neurons, astroglia and microglia a combined protocol for quantitative fluorescence in situ hybridization (qFISH) and immunohistochemistry was used. Length of the telomeric repeat sequence at the end of individual chromosomes was determined by hybridization of human paraffin embedded hippocampal tissue with a synthetic 18-mer peptide nucleic acid (PNA) -probe with a (CCCTAAA)_3_ sequence that was directly conjugated with the fluorescent dye Cy3 (TelC-Cy3, F1002, Panagene). Procedures were performed as described previously [64] with minor modifications.

Paraffin embedded human hippocampal tissue was cut in 5 μm sections (Leica RM2165 microtome). Slides were de-paraffinized with xylene, rehydrated with ethanol and after two washes in phosphate-buffered saline (PBS) placed in citrate buffer (pH 6) in a steamer for 20 minutes. After cooling at room temperature (RT) sections were digested with acidified pepsin (200 mg Pepsin, 100ml H_2_O, 168 μl 37% hydrogen chloride (HCl) for 10 minutes at 37°C, followed by three 5 min washes in PBS. Then slides were ethanol-dehydrated at RT for 5 min and covered with telomere probe hybridization mix [250 μl final volume: 2.5 μl 1M Tris-HCl pH 7.2, 21.4 μl MgCl_2_-buffer pH7.0 (25mM MgCl_2_, 9 mM citric acid, 82 mM Na_2_HPO_4_), 175 μl deionized formamide, 12.5 μl 10% (w/w) blocking reagent, 5 μl PNA Cy3-telomere probe (25μg/ml), 33.6 μl H_2_O]. After 3 min of denaturation at 80°C, slides were incubated at room temperature (RT) for 2h in a wet chamber. Then they were washed two times 5 min with TBS-Tween 1% followed by two 3 min washes with PBS. To avoid unspecific binding slides were incubated with blocking solution (PBS, 10% goat serum, 0.01%Tween) at RT for 60 min followed by two 5 min washes with PBS. For cell specific measurement of telomere length sections were incubated with the respective primary antibody overnight in a wet chamber. Primary antibodies were used as follows: rabbit polyclonal anti-GFAP (ab7260, Abcam, 1/1000), rabbit polyclonal anti-Fox3/NeuN (ab104225, Abcam, 1/500) and rabbit polyclonal anti-IBA-1, Rabbit (Wako, 1/1000). Slides were washed twice for 5 min in PBS and incubated with FITC-conjugated goat anti-rabbit IgG secondary antibody (Jackson Immuno Research, 1/500) for 1 h at RT in a wet chamber. Then slides were washed twice in PBS for 5 min followed by mounting in DAPI mounting solution (Vectashield)

### Assessment of telomere length in tumor cells lines for the conversion of fluorescence intensity into kilobases

One methodological limitation in the fluorometric measurement of telomere length is in the semi-quantitative assessment of fluorescence intensities. To overcome this limitation, we applied a new approach in the investigation of telomere length by creating a conversion factor to calculate TFI into kb length. For this, we used we used five cells lines with defined and stable telomere length (TL) to generate a standard curve to calculate cell type specific TL of each individual (Supplemental Table 2). TL of the appropriate cells was assessed by Southern blot analysis as described previously [65]. All five cells lines were qFISH analyzed together with tissue of ALS-patients and control individuals, which were not processed with citrate buffer and pepsin digestion. Cells lines were thawed on ice and approximately 1×10^5^ cells were used for TL analysis. Cells were washed, dehydrated and hybridized as described above. Then slides were washed twice for 30 min with formamid wash buffer (70 ml Formamide, 1 ml Tris (1M) p*H*7.2, 1 ml BSA (10%), 28 ml H_2_O) followed by three 5 min washes with TBS-Tween 1% and two 5 min washes with PBS. Then slides were mounted in DAPI mounting solution (Vectashield).

### Measurement of telomere fluorescence intensity

Quantification of TFI was performed on Cy3-fluorescence images captured at a magnification of 1000x with the software TFL-TeloV2 [66]. For each individual, TFI was measured in 100 neurons, astroglia and microglia respectively. Analysis was performed on,a total of two sections. The same procedure was applied for the cell lines.

### Calculation of telomere length

For the cell-type-specific calculation of TL in hippocampal ALS- and control- tissue, TFI was measured like described above. TFIs of all five cell lines were used to calculate a conversion factor for the calculation of TFI into kilobases. Linear regression analysis was used to form a linear equitation.

### Statistical analysis

Analysis of disease onset, age of first paresis and survival of G4 *mTerc^−/−^*;*SOD1^G9A3^-* and hemizygous *SOD1^G93A^*—mice were performed using the log-rank test. Statistical analysis was performed using GraphPad Prism software, version 5.04.

Statistical analysis of celltype-specific-TL between ALS-patients and control-individuals was accomplished using the statistical software R package (version 3.2, http://www.r-project.org). P-values are sex- and age- adjusted based on an appropriate multiple linear regression model.

## SUPPLEMENTAL DATA FIGURE, TABLES


