# Supplementary material for: Telomere shortening leads to earlier age of onset in ALS mice
Source: Aging (Albany NY). 2016 Feb 24;8(2):382–91. doi: 10.18632/aging.100904 (PMC4789589; doi:10.18632/aging.100904)
Supplement: Supplementary file 1 [file aging-08-382-s001.pdf]

SUPPLEMENTAL DATA

mTerc$^{+/-}$ *x mTerc*$^{+/+}$*;SOD1*^{G93A}

↓

mTerc$^{+/-}$ *x mTerc*$^{+/-}$*;SOD1*^{G93A}

↓

step 1 *G1 mTerc*$^{-/-}$ *x G1 mTerc*$^{-/-}$*;SOD1*^{G93A}

↓

step 2 *G2 mTerc*$^{-/-}$ *x G2 mTerc*$^{-/-}$*;SOD1*^{G93A}

↓

step 3 *G3 mTerc*$^{-/-}$ *x G3 mTerc*$^{-/-}$*;SOD1*^{G93A}

↓

G4 mTerc$^{-/-}$*;SOD1*^{G93A}

Supplemental Figure 1.

Supplemental Table 1.

Characteristics of human cases

ID	gender	age	cause of death
ALS #1	m	66	pneumonia, coronary heart disease
ALS #2	m	50	ALS
ALS #3	m	69	respiratory insufficiency in ALS
ALS #4	m	75	N/K
ALS #5	m	25	N/K
ALS #6	m	84	chronic heart insufficiency, coronary heart disease
ALS #7	m	43	ALS, pneumonia
ALS #8	m	68	bronchopneumonia
ALS #9	m	74	respiratory insufficiency in ALS
ALS #10	m	63	lymphoplasmocytic lymphoma
ALS #11	f	56	N/K
**ID**	**gender**	**age**	**cause of death**
Control #1	m	45	myocardial infarction, coronary heart disease
Control #2	m	53	pneumonia
Control #3	m	56	chronic myelogenous leukaemia
Control #4	m	58	pulmonary embolism
Control #5	m	46	pulmonary hypertonia, tricuspid insufficiency
Control #6	m	80	acute heart failure, coronary heart disease
Control #7	m	62	carcinoma
Control #8	f	68	liver cirrhosis, hepatitis
Control #9	m	57	myocardial infarction, lung emphysema
Control #10	m	64	myocardial infarction, coronary heart disease
Control #11	m	53	myocardial infarction, coronary heart diesease
Control #12	m	59	liver cirrhosis
Control #13	f	67	multi-organ failure, coronary heart disease

N/K= not known; ALS = Amyotrophic lateral sclerosis

Supplemental Table 2.

Telomere length of human cell lines			
**Cell line**	**Mean telomere length (kb)**	**Origin**	**Company**
HeLa 229	2.77	cervical cancer	ATCC® CCL-2.1
HCT116	3.6	colon carcinoma	ATCC® CCL-247
293THEK	7.27	embryonic kidney	ATCC® PTA-5554
PBMC	10.1	peripheral blood	ATCC® CRL-11968
IMR90h TERT	10.69	fetal lung	ATCC® CCL-186
kb = kilobases			
